# Retraction Note: Birinapant sensitizes platinum-resistant carcinomas with high levels of cIAP to carboplatin therapy

**DOI:** 10.1038/s41698-021-00217-9

**Published:** 2021-08-09

**Authors:** V. La, R. Fujikawa, D. M. Janzen, M. Nunez, L. Bainvoll, L. Hwang, K. Faull, G. Lawson, S. Memarzadeh

**Affiliations:** 1 Department of Obstetrics and Gynecology, David Geffen School of Medicine, University of California Los Angeles, Los Angeles, CA 90095 USA; 2 Department of Psychiatry and Biobehavioral Sciences, David Geffen School of Medicine, University of California Los Angeles, Los Angeles, CA 90095 USA; 3 Pasarow Mass Spectrometry Laboratory, University of California Los Angeles, Los Angeles, CA 90095 USA; 4 Semel Institute for Neuroscience and Human Behavior, University of California Los Angeles, Los Angeles, CA 90095 USA; 5 Office of Laboratory Animal Care, University of California Berkeley, Berkeley, CA 94720 USA; 6 UCLA Eli and Edythe Broad Center of Regenerative Medicine and Stem Cell Research, University of California Los Angeles, Los Angeles, CA 90095 USA; 7 Molecular Biology Institute, University of California Los Angeles, Los Angeles, CA 90095 USA; 8 Department of Biological Chemistry, David Geffen School of Medicine, University of California Los Angeles, Los Angeles, CA 90095 USA; 9 The VA Greater Los Angeles Healthcare System, Los Angeles, CA 90073 USA

Retraction of: *npj Precision Oncology* 10.1038/s41698-017-0008-z, published online 03 April 2017

This Article^1^ has been retracted by the Authors because concerns have been raised regarding the identity of the cell lines used for the experimental work. Specifically, re-analysis showed that the S1GODL xenografts matched STR profiles of Ovcar-3 cells, and review of raw flow cytometry data was found to be discordant with presented data. In light of these findings, the identity of the cells within the experimental groups in the manuscript is uncertain, and the conclusions of the work are not reliable.

Authors Vincent La, Rachel Fujikawa, Deanna Janzen, Miguel Nunez, Lin Hwang, Kym Faull, Greg Lawson, and Sanaz Memarzadeh agree with this attraction. Liat Bainvoll did not respond to correspondence from the Publisher about this retraction.
